# Infant excitation/inhibition balance interacts with executive attention to predict autistic traits in childhood

**DOI:** 10.1186/s13229-022-00526-1

**Published:** 2022-12-08

**Authors:** Virginia Carter Leno, Jannath Begum-Ali, Amy Goodwin, Luke Mason, Greg Pasco, Andrew Pickles, Shruti Garg, Jonathan Green, Tony Charman, Mark H. Johnson, Emily J. H. Jones, the EDEN, Grace Vassallo, Emma Burkitt-Wright, Judith Eelloo, D Gareth Evans, Siobhan West, Eileen Hupton, Lauren Lewis, Louise Robinson, Angus Dobbie, Ruth Drimer, Helen Bethell, Rachel Jones, Susan Musson, Catherine Prem, Miranda Splitt, Karen Horridge, Diana Baralle, Carolyn Redman, Helen Tomkins, Ankita Bhojwani, Shannon Connelly, Francesca Conti, Beth Evans, Meg Jackson, Emily Powell, STAARS Teams, Mary Agyapong, Tessel Bazelmans, Leila Dafner, Mutluhan Ersoy, Teea Gliga, Rianne Haartsen, Hanna Halkola, Alexandra Hendry, Rebecca Holman, Sarah Kalwarowsky, Anna Kolesnik, Nisha Narvekar, Chloë Taylor

**Affiliations:** 1 Institute of Psychiatry, Psychology and Neuroscience, King’s College London, London, UK; 2 Centre for Brain and Cognitive Development, Department of Psychological Sciences, Birkbeck, University of London, London, UK; 3 Faculty of Biological Medical and Health Sciences, University of Manchester, Manchester, UK; 4 Child and Adolescent Mental Health Services, Royal Manchester Children’s Hospital, Central Manchester University Hospitals NHS Foundation Trust, Manchester Academic Health Sciences Centre, Manchester, UK; 5 Department of Psychology, University of Cambridge, Cambridge, UK

**Keywords:** Infants, Autism, ADHD, NF1, E/I balance, Executive functioning

## Abstract

**Background:**

Autism is proposed to be characterised by an atypical balance of cortical excitation and inhibition (E/I). However, most studies have examined E/I alterations in older autistic individuals, meaning that findings could in part reflect homeostatic compensation. To assess the directionality of effects, it is necessary to examine alterations in E/I balance early in the lifespan before symptom emergence. Recent explanatory frameworks have argued that it is also necessary to consider how early risk features interact with later developing modifier factors to predict autism outcomes.

**Method:**

We indexed E/I balance in early infancy by extracting the aperiodic exponent of the slope of the electroencephalogram (EEG) power spectrum (‘1/f’). To validate our index of E/I balance, we tested for differences in the aperiodic exponent in 10-month-old infants with (*n* = 22) and without (*n* = 27) neurofibromatosis type 1 (NF1), a condition thought to be characterised by alterations to cortical inhibition. We then tested for E/I alterations in a larger heterogeneous longitudinal cohort of infants with and without a family history of neurodevelopmental conditions (*n* = 150) who had been followed to early childhood. We tested the relevance of alterations in E/I balance and our proposed modifier, executive attention, by assessing whether associations between 10-month aperiodic slope and 36-month neurodevelopmental traits were moderated by 24-month executive attention. Analyses adjusted for age at EEG assessment, sex and number of EEG trials.

**Results:**

Infants with NF1 were characterised by a higher aperiodic exponent, indicative of greater inhibition, supporting our infant measure of E/I. Longitudinal analyses showed a significant interaction between aperiodic slope and executive attention, such that higher aperiodic exponents predicted greater autistic traits in childhood, but only in infants who also had weaker executive functioning abilities.

**Limitations:**

The current study relied on parent report of infant executive functioning-type abilities; future work is required to replicate effects with objective measures of cognition.

**Conclusions:**

Results suggest alterations in E/I balance are on the developmental pathway to autism outcomes, and that higher executive functioning abilities may buffer the impact of early cortical atypicalities, consistent with proposals that stronger executive functioning abilities may modify the impact of a wide range of risk factors.

**Supplementary Information:**

The online version contains supplementary material available at 10.1186/s13229-022-00526-1.

## Introduction

Autism has been proposed to be characterised by atypical balance of excitation to inhibition in the brain (E/I balance) [1–3], but the vast majority of studies have been conducted with older individuals with an established diagnosis. Understanding the significance of alterations to E/I balance in the mature brain is made challenging by homeostatic compensation and developmental interactions with later emerging characteristics [4]. Since genes linked to autism show peak expression prenatally [5], it is critical to examine E/I alterations early in development before symptoms emerge and to account for individual differences in later maturing brain systems that may modify the developmental impact of early E/I alterations.

A limited number of studies have investigated measures of perceptual processing that may indirectly index E/I balance and/or coordination in infancy. Infants with an older autistic sibling who are later identified as being autistic themselves show heightened pupillary [6] and cortical reactivity [7, 8] in the first year of life, thought to be indicative of an increased E/I ratio. However, novel methods have recently been developed as proxy measures of E/I balance using data from electroencephalography (EEG) recordings [9]. This approach decomposes the periodic (i.e. akin to activity in canonical frequency bands) and aperiodic components of EEG activity, with the aperiodic exponent, or 1/f slope, equivalent to calculating the slope of the power spectrum when measured in log–log space. The slope of the aperiodic component is thought to reflect E/I balance [10], with a steeper slope (a higher aperiodic exponent), reflecting greater inhibition relative to excitation. These non-invasive methods are particularly suitable for developmental studies as EEG assessments are well tolerated by young infants. Aperiodic exponent values appear to track development; they begin to decrease in the first year of life [11] and continue over the lifespan [12, 13], potentially due to cortical maturation and/or increased noise in the ageing brain. Emerging evidence suggests that perturbations in neurodevelopment are associated with alterations to the aperiodic exponent (e.g. schizophrenia [14, 15]), with one recent study of preterm infants reporting higher aperiodic exponents at 9 months associated with higher levels of autistic-type behaviours in childhood [16]. Furthermore, one-month-old infants with a family history of attention deficit hyperactivity disorder (ADHD) exhibit higher aperiodic exponents than infants without a family history of ADHD, whereas stimulant-naïve adolescents with ADHD display lower exponent values than their non-ADHD peers (although opposing adolescent findings are related elsewhere [17]), underscoring the potential for homeostatic compensation and/or developmental specificity of E/I alterations [18].

In addition to delineating alterations in early brain functioning associated with autism outcomes, recent frameworks have noted that the mechanisms that contribute to heterogeneous autism outcomes are unlikely to be simple one-to-one mappings, but instead complex within-individual cascades and interactions [19–21]. The Anterior Modifiers in the Emergence of Neurodevelopmental Disorders (AMEND) model differentiates between disruptions in systems of early-stage processing (primarily those that process sensory and perceptual input, for example sensory habituation and gating), typically present in the first year of life, and later developing modifier factors which alter the capacity of these early-stage processing features to predict neurodevelopmental outcomes [19]. One proposed modifier is infant executive attention, an infant precursor of executive functioning [22, 23]. Stronger executive functioning abilities have been shown to buffer the impact of a variety of known risk factors [24–26], and infant executive functioning-type abilities moderate associations between infant behavioural characteristics and childhood autism traits [27]. Strong executive attention abilities may shift developmental trajectories that began as atypical (by virtue of differences in signal processing as determined by shifts in E/I balance) back towards a more typical outcome, as the developing infant is able to enhance or inhibit processing towards different groups of stimuli [28], promoting learning and adaptive brain development [29]. Accurate models of the developmental processes that characterise neurodevelopmental conditions such as autism need to consider the influence of both early brain differences that may confer risk for atypical outcomes, and consequent interactions with later maturing modifying factors (such as executive attention), which may serve to alter the predictive capacity of these early brain differences.

The first goal of this paper was to validate our proposed metric of E/I balance, the aperiodic exponent of the EEG power spectrum, by applying it to a population of infants with the single-gene disorder neurofibromatosis type 1 (NF1).

**Fig. 1:**
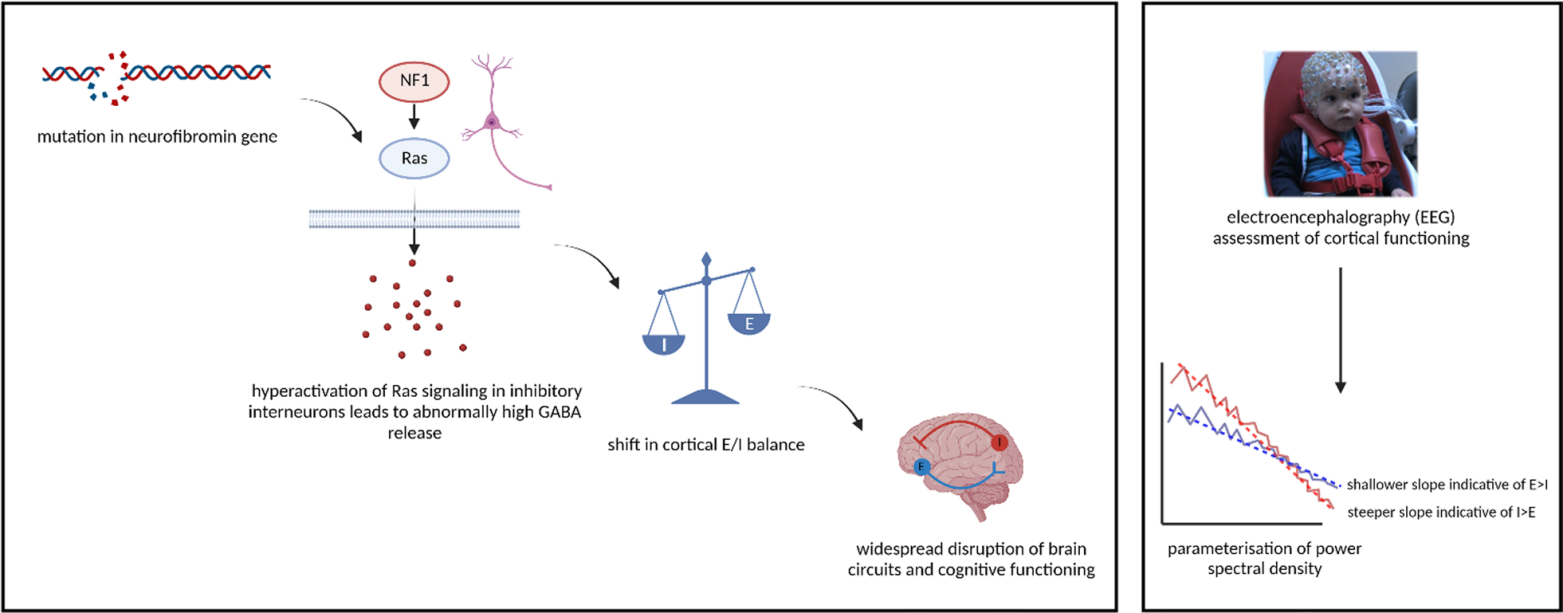
Left: Hypothesised Pathway by Which Mutations in NF1 Gene Alter Cortical Excitation and Inhibition (E/I) Balance and NF1 Behavioural Phenotype. Right: Cortical E/I Balance Can be Measured in Infants by Parameterising EEG Recordings and Extracting the Aperiodic Slope Parameter. Created with BioRender.com

NF1 is thought to be characterised by alterations to E/I balance, with preferential expression of the NF1 gene in inhibitory neurons in both the mouse and human brain [30]. Animal models largely suggest the condition is characterised by increased cortical inhibition as indexed by increases in gamma-aminobutyric acid (GABA) neurotransmission [31–33], one of the primary inhibitory neurotransmitters in the brain (see Fig. 1, left panel for a summary of the hypothesised biological pathway underpinning excess inhibition in NF1 populations); however, some studies in adult humans report the opposite, such that NF1 patients demonstrate decreased inhibition as measured by total GABA concentration [34, 35]. Differing results from animal as compared to human studies may reflect either developmental compensation of early alterations to E/I balance in NF1 patients, or differences in measurement of inhibitory activity (e.g. GABA transmission vs. GABA concentration). Nonetheless, available evidence clearly suggests E/I balance, and in particular activity of the GABA system, is altered in NF1. NF1 is also relevant for understanding pathways to autism because the condition is characterised by an elevated prevalence of autism outcomes (> 40%) [36, 37]). Following [10], we hypothesised that the NF1 group would be characterised by higher aperiodic exponents, indicative of greater inhibition relative to excitation (see Fig. 1, right panel).

Having conducting our validation analyses, we then investigated the main aim of the paper; to test whether alterations in E/I balance interacted with later developing executive attention abilities to predict autistic traits in a larger and more heterogeneous cohort of infants enriched for autism outcomes (with or without a first-degree relative with autism or ADHD [38]) who had been followed longitudinally to childhood. We hypothesised that executive attention would moderate pathways from E/I imbalance to autistic traits, such that a significant association between E/I imbalance and later autistic traits would only be seen in infants with lower executive attention. We selected autistic traits as our outcome given the shift towards dimensional views of the heritable traits contributing to the autism phenotype [39]. Given the high co-occurrence of autism and ADHD [40], overlapping genetic influences [41], evidence that executive functioning is impacted in both conditions [42] and recent reports that aperiodic exponent values may be related to both ADHD family history and diagnosis [18], we also examined longitudinal associations between E/I balance and later ADHD traits. Including ADHD traits as an additional outcome is also relevant to understanding whether phenotypic overlap between these neurodevelopmental conditions can be explained by overlap in neuroendophenotypes that emerge in early infancy [42], and whether executive attention is a moderator for multiple neurodevelopmental outcomes.

## Methods

### Sample

**Fig. 2:**
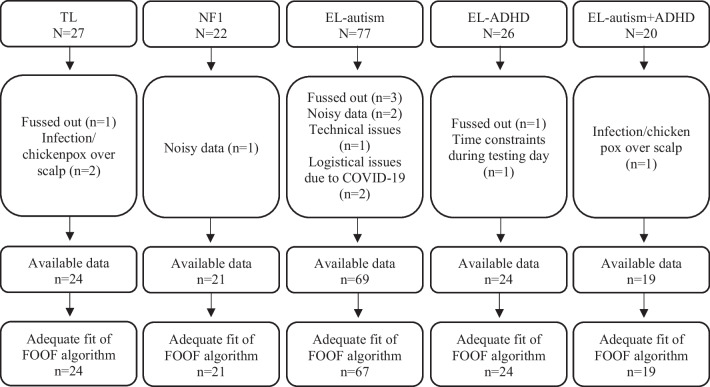
Breakdown of Participant Dropout at 10-Month Visit. EL-ADHD = elevated likelihood for ADHD, EL-autism + ADHD = elevated likelihood for autism and ADHD, EL-autism = elevated likelihood for autism, NF1 = neurofibromatosis type 1, TL = typical likelihood

Infants in the NF1 group were recruited through local medical and genetic centres, and the remaining infants were recruited as part of a longitudinal prospective study (Studying Autism and Attention Deficit Hyperactivity Disorder Risks programme; STAARS) [see [43] for more details]. All infants were born full-term (gestational age 36–42 weeks). At the time of enrolment, none of the infants (aside from those in the NF1 group) had a known medical or developmental condition. Informed written consent was provided by the parent(s) prior to the commencement of the study. The study was approved by the National Research Ethics Service and the Research Ethics Committees of Birkbeck, University of London and King’s College London. All NF1 infants had their diagnosis confirmed via molecular testing of cord blood samples or clinical diagnosis based on NIH consensus criteria [44] and had no other developmental concerns at the time of the visits. The remaining infants were assigned group membership for familial likelihood of autism and ADHD based on information on clinical diagnoses and scores on various screening measures (see Additional file 1 for more information). Infants in the EL-autism group had at least one first-degree relative with a community clinical diagnosis of autism, infants in the EL-ADHD group had at least one first-degree relative with a community clinical diagnosis or probable research diagnosis of ADHD, and infants in the TL group had at least one older sibling with typical development and no known autism or ADHD in first-degree family members (as confirmed through parent interviews regarding family medical history). The final sample included for current analyses includes data from 20 infants with NF1, 67 infants with an elevated likelihood of autism (EL-autism), 24 infants with an elevated likelihood of ADHD (EL-ADHD), 19 infants with an elevated likelihood of both ASD and ADHD (EL-autism + ADHD) and 24 infants with a typical likelihood for autism and/or ADHD (TL). See Table 1 for summary statistics of the included sample and Fig. 2 for a breakdown of participant retention at each time point [adapted from [45]].

**Table 1:** Demographic characteristics of included sample

Mean (SD)	TL	NF1	EL-autism	EL-ADHD	EL-autism + ADHD	Group contrast p value
**10-month visit**						
*n*	24	21	67	24	19	–
Social videos: n retained trials	91.19 (47.84)	87.33 (36.15)	98.34 (44.64)	105.27 (31.72)	100.94 (49.91)	0.63
Non-social videos: n retained trials	95.21 (46.17)	90.94 (36.23)	89.04 (41.46)	95.73 (28.47)	87.27 (39.04)	0.92
Males/females (% female)	13:11 (46%)	9:12 (43%)	34:33 (49%)	13:11 (42%)	11:8 (40%)	0.90
Age in months	10.75 (0.55)	10.84 (0.65)	10.62 (0.49) ^(n=66)^	10.87 (0.93)	10.63 (0.49)	0.36
MSEL ELC	88.71 (12.88)	80.75 (10.65) ^(n=20)^	87.74 (15.56) ^(n=66)^	85.54 (16.12)	82.32 (12.17)	0.22
Average RMSEA	0.07 (0.02)	0.06 (0.02)	0.06 (0.01)	0.06 (0.01)	0.06 (0.01)	0.18
**24-month visit**						
*n*	21	n/a	56	19	15	–
Males/females (% female)	10:11 (52%)	n/a	29:27 (48%)	11:8 (42%)	10:5 (33%)	0.67
Age in months	25.42 (1.26)	n/a	25.92 (1.64)	25.63 (1.29)	25.08 (0.52)	0.17
MSEL ELC	113.29 (18.82)	n/a	99.20 (21.43)	107.95 (22.62)	96.73 (17.70)	0.03
EBCQ executive attention score	4.54 (0.78) ^(n=20)^	n/a	4.19 (0.95) ^(n=53)^	4.12 (0.79) ^(n=15)^	3.92 (0.71) ^(n=12)^	0.24
**36-month visit**						
*n*	16	n/a	54	21	15	–
Males/females (% female)	9:7 (56%)	n/a	25:29 (54%)	11:10 (48%)	10:5 (33%)	0.55
Age in months	37.98 (2.03)	n/a	38.10 (1.33)	38.44 (2.75)	38.04 (1.54)	0.86
MSEL ELC	128.38 (12.20)	n/a	107.06 (19.09) ^(n=53)^	118.67 (19.73)	106.29 (20.60) ^(n=14)^	< 0.001
SRS total	25.13 (9.22)	n/a	43.13 (32.91) ^(n=49)^	33.06 (25.19) ^(n=18)^	65.36 (48.35) ^(n=11)^	< 0.001
SRS t-score	43.56 (3.67)	n/a	50.57 (12.60) ^(n=49)^	46.61 (9.61) ^(n=18)^	59.00 (18.63) ^(n=11)^	< 0.01
CBCL ADHD subscale total	2.72 (2.14)	n/a	4.29 (3.26) ^(n=52)^	4.58 (3.44) ^(n=19)^	6.08 (3.93) ^(n=13)^	0.04
CBCL ADHD subscale t-score	50.89 (1.37)	n/a	54.02 (6.30) ^(n=52)^	54.58 (7.14) ^(n=19)^	58.08 (9.03) ^(n=13)^	0.02

CBCL, Child Behavior Checklist; ECBQ, Early Childhood Behavioral Questionnaire; EL, elevated likelihood; ELC, Early Learning Composite; MSEL, Mullen Scales of Early Learning; SD, standard deviation; and SRS, Social Responsiveness Scale

### Questionnaire measures

Executive attention was measured at 24 months with the Early Childhood Behavioral Questionnaire—Short Version [46], designed to assess temperament in children aged one to three years old. Parents rate how often their child exhibited each behaviour in the previous two weeks scored from 1 (Never) to 7 (Always). For current analyses, we generated a score for executive attention by summing scores on the Inhibitory Control, Attention Shifting and Attention Focusing subscales, each consisting of 12 items, which make up the wider Effortful Control subscale. We excluded the Low-Intensity Pleasure and Cuddliness subscales to ensure measurement was not biased by atypical expressions of affective states [as these may be more prevalent in autistic children; [47, 48]]. Confirmatory factor analysis in the full 24-month sample (*N* = 114) suggested scores from the Inhibitory Control, Attention Shifting and Attention Focusing subscales all significantly loaded on the hypothesised executive attention factor (loadings = 0.69–0.79, all *p*s < 0.001).

Autism traits were measured at 36 months using the Preschool form of the Social Responsiveness Scale—2 [49]. The SRS is designed to measure autistic traits in the general population and consists of 65 items, each rated on a 4-point scale ranging from 1 (Not True) to 4 (Almost Always True).

ADHD traits were measured at 36 months using the Preschool Child Behavior Checklist DSM Attention Deficit/Hyperactivity Problems subscale [50], which comprises six items that measure inattentive and hyperactive behaviours over the past two months. Parents are asked to indicate how well each statement describes their child’s behaviour ranging from 0 (Not True) to 2 (Very True or Often True).

### Experimental stimuli

Infants were shown naturalistic social (women singing) or non-social (toys moving) dynamic videos designed to produce calm attention [51]. Social videos consisted of the face, torso and hands of two women singing nursery rhymes with corresponding gestures. The nursery rhymes were: ‘Hi Baby, Where Are My Eyes?’, ‘Itsy Bitsy Spider’, ‘The Wheels on the Bus’, ‘Twinkle Twinkle Little Star’ and ‘Pat-a-cake’ (played in this fixed order). In the Non-Social video, infant appropriate toys were shown to be moving (e.g. spinning toys in motion, balls popping within a clear plastic toy, balls moving down a chute). There was no social content to these videos. The order of the videos was counterbalanced across infants, and other visual tasks (not reported in this paper) were presented between each block of videos. The videos were presented on a screen with a diagonal size of 23″ (58.42 cm × 28.6 cm, 52° × 26.8°, aspect ratio of 16:9). To ensure that all participants saw the same sized stimuli (in case of technical issues with/changes in the monitor screen over the course of this longitudinal study), we presented the stimuli within a ‘virtual window’ at the following size: a diagonal size of 17″ (34.5 cm × 25.9 cm, 32.1° × 24.4°, with a native resolution of 1280 × 1024 pixels and an aspect ratio of 5:4) and with black borders around the edge of the screen. Stimuli were therefore drawn with an effective display resolution of 37.1 pixels per cm. In order to maintain the source aspect ratio of 16:9 when presented within the ‘virtual window’, all videos were scaled to 32.6 cm × 31 cm (30.4° × 29°, 1210 × 1150 pixels) on screen. Videos were 1 min in length and presented up to 3 times, for a total of 3 min each, interspersed through a longer EEG session.

### EEG acquisition and procedure

EEG was recorded using an EGI (Philips Neuro, Oregon, USA) 128-electrode Hydrocel Sensor Net, online referenced to Cz at 500 Hz. Infants were seated on their caregiver’s lap, 60 cm from a screen. All testing took place in a sound attenuated and electrically shielded room. A video camera situated below the screen used for stimulus presentation recorded the infants’ bodily and facial behaviour.

EEG was bandpass filtered (0.1–100 Hz), and 1-s segmented. Data were manually cleaned in NetStation 4.5 [52]; segments with excessive artefact (e.g. gross motor movement, eye blinks), where infants were not looking at the video, or with > 25 noisy channels were manually excluded. Infants with < 10 artefact-free trials in either condition were excluded (see Additional file 1: Fig. S1). Noisy channels were interpolated from neighbouring channels using spline interpolation. 1-s non-overlapping segments were referenced to the average reference, imported into MATLAB, detrended and subjected to a fast Fourier transform (FFT). Power values were calculated and averaged across artefact-free segments in 1 Hz bins.

### Extraction of E/I metrics from EEG

The fitting oscillations and one over f (FOOOF) algorithm was used to obtain individual aperiodic exponent values [9]. When the power spectrum is plotted on a log–log axis (i.e. power on the y-axis, frequency on the x-axis), the aperiodic exponent is equivalent to the coefficient of a regression line characterising the slope. Aperiodic exponents were estimated for social and non-social videos separately and then averaged. Following previous work with infant samples [11], we parameterised spectra in the frequency range 1–10 Hz to avoid contamination by higher frequency artefacts, and only exponents from model fits with R^2^ ≥ 0.95 were kept for further analysis. Other settings were as follows: peak width limits = 2, 8, maximum number of peaks = 4, peak threshold = 0.1, aperiodic mode = ‘fixed’. Aperiodic exponent values were extracted from frontal, central and posterior regions (frontal = electrodes ﻿2, 3, 4, 11, 19, 23, 26, 9, 10, 18, 22, 15, 16, central = electrodes 36, 104, 30, 7, 106, 105, 31, 37, 80, 87, 55, and posterior = electrodes 52, 62, 92, 61, 77, 78, 53, 86, 60, 67, 72, 85). Comparison of estimated aperiodic components overlaid against input EEG data was visually inspected for each participant. Based on the R^2^ threshold, data from two further EL-autism infants were excluded.

### Statistical analysis

Statistical analyses were conducted in Stata 16 [53]. To minimise the impact of outliers whilst retaining data, aperiodic exponent values were winsorised such that the 5% of the lowest/highest values were recoded to the value of the 5^th^/95th percentile. As there were no differences in aperiodic exponent values between the social and non-social videos (*p* = 0.41), we collapsed values across conditions to maximise the signal-to-noise ratio. First, to validate our metric of E/I balance, we tested for differences in 10-month aperiodic exponent between NF1 and TL infants. We ran a mixed effects model, with region (Fz, Cz, Pz) as a within-subjects factor, 10-month aperiodic exponent as the outcome and the following predictors: NF1 status (present/absent), age in years at 10-month visit, number of EEG trials (averaged between social and non-social videos) and sex. As we did not have any a priori hypotheses for the topography of group differences, NF1 status*region interaction terms were only run if NF1 status effects were seen first. Next, we compared differences in aperiodic exponents between the EL and TL groups, splitting the EL group dependent on the type of familial likelihood status (EL-autism, EL-ADHD). We ran a comparable mixed effects model, but with EL-autism status (present/absent), EL-ADHD status (present/absent), region, age in years at 10-month visit, number of EEG trials (averaged between social and non-social videos) and sex as predictors of 10-month aperiodic exponent values. Secondary models included an interaction between EL-autism and EL-ADHD status, which allowed us to test if there were any additive/protective effects of the combined group. As before, likelihood status*region interaction terms were only run if likelihood status effects were seen first. We re-ran the EL-TL contrast models excluding infants with < 20 EEG trials (*n* = 4) and the pattern of results remained the same. (There were no infants with < 20 EEG trials in the NF1-TL contrast models.) Both the NF1-TL and the EL-TL comparison models were run with restricted maximum likelihood. We use the method outlined in [54, 55] to generate Cohen’s *f*^2^ from mixed effect models and report these for any significant group comparisons. Here, effects are considered small at values around 0.02, medium at values around 0.15 and large at values around 0.35 [56].

To test whether 24-month executive attention moderated associations between early E/I imbalance and later autism and ADHD traits in the combined EL and TL sample, we ran two linear regressions, with 36-month SRS total and CBCL DSM ADHD subscale total score as the outcome, respectively. First, we tested the main effect of 10-month aperiodic exponent (averaged across all three regions). After running main effects, we added 24-month executive attention and an interaction between aperiodic exponent and executive attention as predictors. In all models, we included age in months at 10-month visit, number of EEG trials, sex and likelihood group (EL-autism, EL-ADHD and the interaction of the two) as covariates. The SRS and CBCL were non-normally distributed and therefore square root transformed. As the Breusch–Pagan/Cook–Weisberg test for heteroskedasticity of residuals was significant for the SRS (*p* < 0.01), all analyses with SRS as the outcome were run with robust standard errors. As further robustness checks, we re-ran longitudinal models 1) excluding infants with < 20 trials (*n* = 4), 2) including 10-month head movement (as described in [57]) as an additional covariate to check that global trait-like differences in activity level not captured by individual differences in the number of trials were not contributing to results. This movement variable captures the amount of head movement as measured by an eye tracker during a separate battery of eye-tracking tasks that was administered during the same visit as the EEG assessment and 3) using a modified SRS total which has been proposed to be a more precise measurement of autistic traits as it excludes items which could relate to other co-occurring conditions [58]. We report both unstandardised (b) and standardised (β) coefficients.

## Results

### Validation of E/I metric

**Fig. 3:**
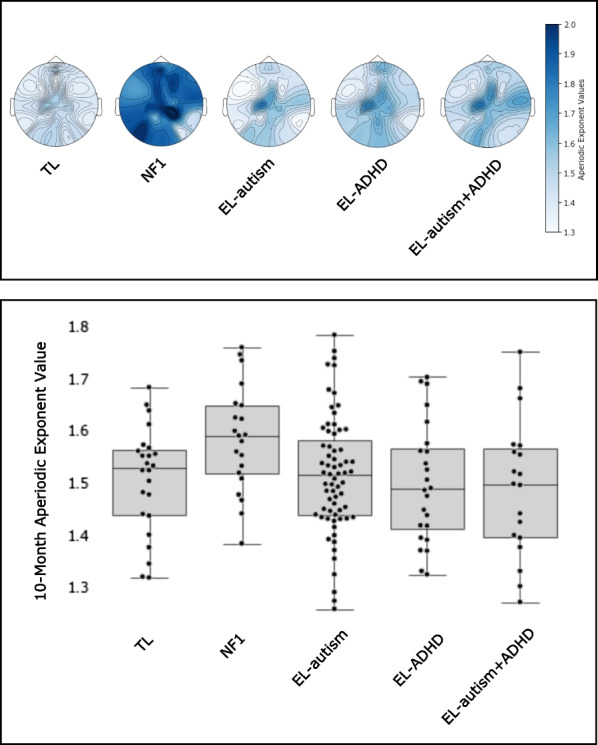
Topographic Distribution (top) and Individual Estimates (bottom) of Aperiodic Exponent Values at 10 Months of Age in Infants with Typical Likelihood (TL) of Neurodevelopmental Conditions, Infants with Neurofibromatosis Type 1 (NF1), Infants with Elevated Likelihood of Autism Outcomes (EL-autism), Infants with Elevated Likelihood of ADHD Outcomes (EL-ADHD) and Infants with Elevated Likelihood of Autism and ADHD Outcomes (EL-autism + ADHD)

Results indicated higher aperiodic exponents in infants with NF1 (*b* = 0.07, 95% CIs [0.02, 0.13], *p* = 0.01; marginal predicted means for the NF1 group = 1.58, 95% CIs [1.54, 1.62] and for the TL group = 1.50, 95% CIs [1.47, 1.53]; see Fig. 3). Calculation of Cohen’s *f*^2^ for the NF1 coefficient suggested a small effect size (0.04). We also saw a significant effect for number of trials, such that infants with more trials showed smaller exponents (*b* = − 0.01, 95% CIs [− 0.01, 0.01], *p* < 0.01) (but trial numbers did not differ by group, see Table 1). Exponent values did not vary by region (combined test of regional effects: χ^2^(2) = 2.12, *p* = 0.35), sex (*b* = 0.01, 95% CIs [− 0.05, 0.06], *p* = 0.92) or age (*b* = − 0.22, 95% CIs [− 0.81, 0.37], *p* = 0.46). We did not find evidence for topographical specificity of NF1 effects, suggesting that effects were not localised to a particular region (combined NF1*region interaction term; χ^2^(2) = 0.34, *p* = 0.84).

### Prediction of childhood autistic traits

We found no contemporaneous effect of elevated familial likelihood for neurodevelopmental outcomes on aperiodic exponent values, with no difference between the TL and the EL-autism (*b* = 0.01, 95% CIs [− 0.04, 0.04], *p* = 0.89) or EL-ADHD group (*b* = − 0.01, 95% CIs [− 0.05, 0.03], *p* = 0.54). Similarly, the EL-autism*EL-ADHD interaction term was non-significant (*b* = − 0.03, 95% CIs [− 0.11, 0.05], *p* = 0.49).

**Fig. 4:**
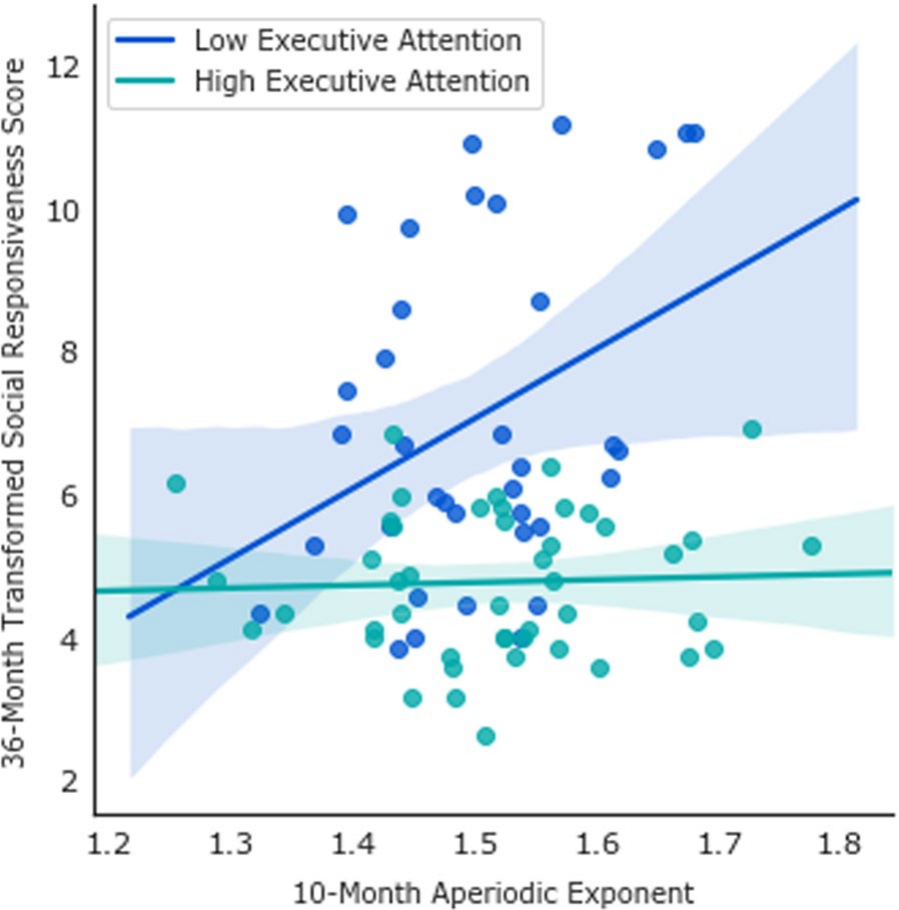
Associations between 10-month Markers of Excitation/Inhibition Balance and Childhood Autism Traits Are Dependent Upon Executive Attention Abilities

Longitudinal analyses showed that there was a direct effect of EL-autism status (*b* = 1.57, 95% 95% CIs [0.72, 2.42], *p* < 0.001, *β* = 0.34), such that infants with a familial history of autism were characterised by higher autistic traits at 36 months. No other direct effects were significant (see Table 2 for full output). In interaction models, the 10-month aperiodic exponent*24-month executive attention interaction was significant (*b* = − 3.99, 95% CIs [− 7.39, − 0.60], *p* = 0.02, *β* = − 2.68), and when associations were broken down by median executive attention score, analyses showed the 10-month aperiodic exponent was significantly associated with SRS scores in the low (*n* = 36; *b* = 12.19, 95% CIs [3.69, 20.70], *p* = 0.007, *β* = 0.44) but not high (*n* = 45; *b* = 0.67, 95% CIs [− 2.92, 4.25], *p* = 0.709, *β* = 0.07) executive attention group (see Fig. 4). Summary statistics split by median grouping are presented in Additional file 1: Table S1, which highlight that moderation effects were not simply due to lack of variability in the predictor in the low executive attention group. When ADHD traits were entered as the outcome, we found no significant main or interactive effect (see Table 2). Results were unchanged when infants with < 20 EEG trials (*n* = 4) were excluded or when 10-month head movement was included as a covariate. Longitudinal models using the modified SRS total results also remained substantially similar (see Additional file 1: Table S2).

**Table 2:** Associations between infant metrics of E/I balance and neurodevelopmental traits in toddlerhood

Predictor	36-Month Autism Traits (SRS Total Score)				36-Month ADHD Traits (CBCL ADHD Subscale)			
	*b*	95% CIs	*β*	*p*	*b*	95% CIs	*β*	*p*
**Model 1: Main effects**								
10-month aperiodic exponent	2.49	[− 2.43, 7.41]	0.11	0.32	0.66	[− 1.25, 2.56]	0.07	0.50
EL-autism status	1.57	[0.72, 2.42]	0.34	< 0.001	0.39	[− 0.01, 0.80]	0.20	0.06
EL-ADHD status	0.89	[− 0.14, 1.93]	0.19	0.09	0.35	[− 0.07, 0.77]	0.17	0.10
EL-ADHD + autism status	− 0.78	[− 2.86, 1.31]	− 0.14	0.46	0.30	[− 0.55, 1.14]	0.12	0.49
Sex	− 0.74	[− 1.60, 0.12]	− 0.16	0.09	− 0.31	[− 0.69, 0.06]	− 0.17	0.10
Age in years at 10-month visit	3.61	[− 4.28, 11.49]	0.08	0.37	0.64	[− 3.01, 4.28]	0.03	0.73
Number of EEG Trials at 10-month visit	− 0.01	[− 0.01, 0.01]	− 0.06	0.53	− 0.01	[− 0.01, 0.01]	− 0.13	0.21
**Model 2: Interaction effects**								
24-month executive attention	4.46	[− .78, 9.70]	1.87	0.09	− 2.46	[− 4.95, 0.04]	− 2.21	0.05
10-month aperiodic exponent*24-month executive attention	− 3.99	[− 7.39, − 0.60]	− 2.68	0.02	1.12	[− 0.51, 2.75]	1.60	0.18

CBCL, Child Behavior Checklist; EL, elevated likelihood; SRS, Social Responsiveness Scale; *b*, unstandardised coefficient; *β*, standardised coefficient

## Discussion

The current study 1) supports our proposed metric of E/I balance in the first year of life by demonstrating differences in our EEG signal in a population of infants known to be characterised by alterations in E/I balance (infants with NF1) and 2) tested whether longitudinal associations between infant E/I alterations and later autism traits were moderated by executive attention in a sample enriched for neurodevelopmental outcomes who had been followed to childhood. Results showed infants with NF1 had a higher aperiodic exponent than typically developing infants, suggestive of greater inhibition relative to excitation. In line with our predicted hypotheses, in the sample of infants enriched for neurodevelopmental outcomes, executive attention moderated the association between E/I balance at 10 months and autism traits at 36 months, such that an association between higher aperiodic exponents (indicative of lower E/I) and higher levels of autism traits was only found in infants who had lower levels of executive attention at 2 years. We found no associations between E/I balance and familial likelihood for ADHD or later ADHD traits, and no evidence of moderation, suggesting some specificity to autism outcomes.

### Infant EEG metrics of E/I as potential translational tool

Our analyses demonstrate infants with NF1 are characterised by alterations in E/I balance early in the developmental pathway, specifically increased inhibition relative to excitation (although it should be held in mind, the effect size was small). This is in line with evidence of preferential NF1 gene expression in inhibitory neurons [30] and animal models that find NF1 is characterised by increases in cortical inhibition [31–33] (however, our results contrast with studies of GABA concentration in adults with NF1 that report decreased inhibition; [34, 35]). Thus, results suggest 1/f-type EEG signals may be a useful translational marker of E/I balance for use in cross-species research. By first testing our E/I metric in a group of infants with a known genetic disruption that preferentially impacts inhibitory interneurons, results suggest that increased inhibition at a molecular level is reflected in characteristics of the EEG power spectral density, specifically the aperiodic exponent. To our knowledge, there currently exist few validated indicators of E/I balance which are suitable for use in developmental populations. Our approach highlights the value in selecting populations with a known genetic alteration that are well characterised in terms of the impact of genetic alterations on molecular and cortical signalling pathways, but also experience autism outcomes at higher rates. These more narrowly defined and homogenous populations can be used to validate novel metrics of cortical functioning before investigating the impact of early differences in neural functioning on later developing autism characteristics in more heterogeneous samples of infants who had been followed up longitudinally. Establishing alterations in E/I balance to precede and predict autism trait emergence is crucial to understanding the aetiological relevance of these neurobiological characteristics with regard to autism outcomes.

### Prediction of autistic traits depends on interactions between risk and resilience factors

We did not find contemporaneous differences at 10 months of age in E/I balance between infants with and without family history of neurodevelopmental disorders. We suspect this is likely due to the increased heterogeneity within the family history group as compared to a genetic risk group like NF1 (although we highlight that genetic conditions such as NF1 still show significant heterogeneity in underlying biology and phenotypic presentation), both in terms of the fact that within infants with a family history of neurodevelopmental disorders, only around ~ 20% will go onto be identified as having autism themselves [38], but also that there are likely multiple developmental pathways to phenotypically similar neurodevelopmental outcomes (i.e. equifinality). Delineation of causal paths from genetic liability to neurodevelopmental outcomes requires parsing of said heterogeneity. Some have taken a ‘neurobiology-first’ approach, using unsupervised learning approaches to identify different neurotypes of autism [59]. Here, we take a ‘genetic-first’ approach, using a group at enhanced likelihood for neurodevelopmental outcomes with a known genetic disruption and well-investigated molecular profile to inform the identification of risk features, before testing their interactions with resilience factors in a more genetically heterogeneous cohort. The current approach may be especially valuable to identify neurodevelopmental risk factors that are present early in the infant period, as currently infant samples are not of the same magnitude as adult samples, which will limit the number of neurotypes statistical models can robustly identify and validate. Furthermore, genetic-first approaches have the advantage of resulting in more interpretable patterns as they benefit from existing knowledge of associated biological profiles—solely relying on unsupervised data-driven approaches can often lead to difficulties in terms of identification of the underlying biological mechanisms that underpin different subgroups.

In line with our predictions, we found that higher aperiodic exponent values (indicative of increased inhibition) in infancy were associated with greater autism traits in childhood only in infants who also had lower executive attention (a predictor of later executive functioning abilities; [22]). Findings are in line with frameworks of neurodevelopmental conditions which highlight the need to consider interactions between early-stage neurobiological differences and later developing characteristics [19] and suggest that later developing modifier factors may alter the capacity of early-stage processing features to predict neurodevelopmental outcomes [60] and may help to understand variability in the developmental expression of the autism phenotype [20]. These frameworks have important implications for the future of neurodevelopmental research; if neurodevelopmental outcomes such as autistic traits are the product of multiple interacting brain systems, it is unlikely that specific and localisable brain differences will be associated with the outcome of interest in all individuals (i.e. modifier factors may mask associations between risk factors and outcome). Thus, research that seeks to delineate the aetiological pathways to autism outcomes needs to consider not only the role of risk factors, but also the influence of opposing modifier mechanisms. Characterising endogenous modifier factors will also inform the design and evaluation of intervention strategies for autistic populations.

Results suggest that differences in early perceptual/sensory processing systems (as indexed by alterations to cortical E/I balance; see [61] for a review of the role of E/I activity in sensory processing) are more likely to increase the likelihood for autism outcomes if the infant’s emergent executive functioning skills are relatively weak. Infants with higher executive attention abilities may be better able to select and attend to relevant parts of their environment, thereby prompting adaptive learning during key periods and minimising the impact of early cortical atypicalities on cognitive development, which may nudge their developmental trajectories towards a more neurotypical outcome [29]. The idea that executive functioning abilities may act as modifiers of early atypicalities is also supported by the fact that the cortical regions which support executive functioning abilities, namely the prefrontal cortex, mature relatively later in development [62] (and thus can be viewed as developing somewhat independently from primary sensory/motor systems), are seen to act as top-down modulators of other domains of cognition [28] and may play a role in orchestrating the functional organisation of other brain regions during development [63]. Combining paradigms such as the one included at present (e.g. restful attention) with paradigms specifically designed to measure evoked neural response to different sensory inputs is necessary to understand how differences in E/I balance modulates processing of incoming stimuli, and how in turn this may increase the likelihood of autistic traits. The buffering role of executive functioning has been shown for a variety of psychiatric risk factors [24–26], and early autism-like behaviours in 14-month-old infants only predict autism behaviours in middle-childhood in infants who also have lower regulatory function (another infant precursor to executive functioning) [27]. We did not find a similar pattern of results for ADHD traits, suggesting some specificity in the role of early alterations in E/I balance (although see [18]). The fact we included fewer ADHD probands, and that ADHD-type behaviours may be less clear at such a young age, all could have both impacted the ability to detect effects (although we note the distribution of t-scores for ADHD traits at 36 months was comparable to that of autistic traits). Finally, we highlight the potential relevance of our findings to the concept of camouflaging in the autism literature [64, 65]. Our proposed moderator of executive functioning-type abilities may also in part be indexing certain components of camouflaging (e.g. following and imitating others, also referred to as masking), leading to fewer behaviourally manifested autism traits. Whether this type of camouflaging behaviour is present in such young children, and whether executive functioning-type abilities are relevant for understanding camouflaging propensity and aptitude, requires further research.

Interestingly, the current association between *decreased* excitation (relative to inhibition) as a predictor of later autism traits opposes suggestions of increased cortical excitation in autistic populations [1], although is comparable to recent work in infants born preterm, which report higher aperiodic exponents at ten months of age are associated with higher autistic-type behaviours at age three [16]. One factor to consider is the dynamic nature of E/I balance across the lifespan [66], underscoring the need for developmental studies in the first years of life. For example, reports of reduced inhibitory neurotransmitter GABA in older autistic individuals [67] could in part reflect homeostatic compensation to excess cortical inhibition at earlier stages [4]. However, studies conducted with infants of a similar age find neural alterations suggestive of increased excitation/decreased inhibition are associated with later autism outcomes [e.g. repetitive suppression; [7, 8]]. One explanation is differences in paradigms: here, we use videos designed to elicit ongoing restful attention, whereas others examine event-related responses. Furthermore, some argue that inhibition, rather than excitation, is crucial for evoked oscillatory responses as this allows for selective synchronous neural activity [68, 69]. It is also possible that reductions in repetition suppression do not represent decreased inhibition but instead subtle variations in the neural populations activated by repeated tones (which would not be detected by comparison of the magnitude of evoked response), which potentially indicate greater precision and thus greater inhibition.

### Limitations

We relied on parent report of children’s executive functioning abilities and used a composite score from a more general questionnaire that indexes a range of infant behaviours. Future work would benefit from more specific and objective measures of executive functioning, for example, brain-level assessment of prefrontal cortex activity [e.g. [70]]. This would allow evaluation of real-time interactions between prefrontal cortex activity and sensory processing, which is necessary to further understand how individual differences in prefrontal activity and executive functioning-type abilities moderate processing of incoming sensory information and/or the behavioural response to sensory processing differences. Similarly, we used a measure of autistic traits as our outcome of interest, this is not directly comparable to a diagnosis, and we highlight that only a small percentage of the current sample went on to receive a research diagnosis of autism at 36 months (around 10%). Future work in samples with sufficient statistical power is needed to test whether 1/f-type signals and their interaction with executive function are useful indicators of later diagnostic status. We reiterate that the effect size of differences in E/I balance between the NF1 and TL groups was small and therefore should be interpreted accordingly. We also highlight the association between number of trials and aperiodic exponent, which requires further investigation to understand the impact of fewer trials on EEG signal estimation and the minimum number of trials required for robust measurement of 1/f-type signals, but suggests future studies of 1/f-type signals should include trial number as a covariate. Additionally, the impact of neurodevelopmental heterogeneity on cortical functioning in NF1 populations (e.g. additional autism or ADHD diagnoses, other relevant characteristics) is not well characterised and is an important step for future work. Finally, our longitudinal analyses mostly included children with IQ in the average range; future work is required to understand the relevance of alterations in E/I balance and executive functioning-type abilities for neurodevelopmental outcomes in infants with a wider range of cognitive ability (e.g. including individuals with intellectual disabilities).

## Conclusions

Results suggest 1/f-type EEG signals may be a useful translational marker of E/I balance for use in developmental studies. Findings support our hypothesis that emerging executive functioning abilities buffer the developmental impact of early life atypicalities in E/I balance with regard to autism outcomes. Results highlight the need for prospective hypothesis-driven and biologically informed research in order to unpick causal paths and model the interactive effects of different features which lie on the path to autism outcomes.

## Supplementary Information

**Additional file 1:** Supplementary Materials.

## Data Availability

The datasets analysed during the current study are subject to the BASIS data sharing policy (see http://www.basis-network.org). The scripts for the extraction of the aperiodic component and the statistical analysis can be found at OSF https://doi.org/10.17605/OSF.IO/2VWXY

## Abbreviations

ADHD: Attention deficit hyperactivity disorder
CBCL: Child Behavior Checklist
ECBQ: Early Childhood Behavioral Questionnaire
EEG: Electroencephalogram
E/I: Excitation/inhibition
EL-ADHD: Elevated likelihood for ADHD outcomes
EL-autism: Elevated likelihood for autism outcomes
ELC: Early Learning Composite
GABA: Gamma-aminobutyric acid
MSEL: Mullen Scales of Early Learning
NF1: Neurofibromatosis type 1
SRS: Social Responsiveness Scale
TL: Typical likelihood
