# Supplementary material for: Infant excitation/inhibition balance interacts with executive attention to predict autistic traits in childhood
Source: Mol Autism. 2022 Dec 8;13:46. doi: 10.1186/s13229-022-00526-1 (PMC9733024; doi:10.1186/s13229-022-00526-1)
Supplement: Supplementary file 1 — Additional file 1: Supplementary Materials. [file 13229_2022_526_MOESM1_ESM.docx]

**Infant Excitation/Inhibition Balance Interacts with Executive Attention to Predict Autistic Traits in Childhood**

**Supplementary Materials**

*Sample Characterisation*

Infants in the EL-autism group had at least one first-degree relative with a community clinical diagnosis of autism, which was complemented where possible with parent-report measures (the Development and Wellbeing Assessment [DAWBA; 1], the Social Communication Questionnaire [SCQ; 2]).

Infants in the EL-ADHD group had at least one first-degree relative with a community clinical diagnosis or probable research diagnosis of ADHD. Parents were also asked if they had any concerns about ADHD within the family. For those who reported concerns, but the first-degree relative didn’t have a diagnosis of ADHD, screening questionnaires were used to examine the probable existence of ADHD. For siblings aged less than 6 years, a shortened version of the Conners Early Childhood form was used [3]. For siblings 6 years or older, a shortened version of the Conners 3 was used [4]. Thresholds for inclusion in the ADHD category were the presence of 6 ADHD symptoms on either the hyperactivity/impulsivity or inattention scale, and a positive score on the impairment scale. For parents, a shortened version of the Conners Adult ADHD Rating Scale [5] was used. Thresholds for inclusion were the presence of 5 ADHD symptoms on either the hyperactivity/impulsivity or inattention scale as per updated DSM-5 guidelines. This additional screening was undertaken as had we applied the clinical diagnosis rule for ADHD likelihood status as we had for autism likelihood, this may have risked under-identification of ADHD.

*Supplementary Table 1. Group Differences on Key Variables Based on Moderator (Median Split on Executive Attention)*

| **Mean (SD; range)** | **Low Executive Attention (n=49)** | **High Executive Attention (n=52)** |
| --- | --- | --- |
| 10-month aperiodic exponent | 1.51 (.10; 1.27-1.75) | 1.52 (.12; 1.26-1.78) |
| EL-autism status (absent/present, % present) | 15:34 (69%) | 20:31 (60%) |
| EL-ADHD status (absent/present, % present) | 34:15 (31%) | 39:12 (24%) |
| Sex (M:F, % female) | 31:18 (37%) | 23:29 (56%) |
| Age in months at 10-month visit | 10.70 (.58; 9.27-12.70) | 10.72 (.61; 9.63-12.80) |
| Number of EEG Trials at 10-month visit | 91.36 (39.35; 16.05-160.50) | 94.37 (42.05; 12.39-157.33) |
| 24-month executive attention score | 3.51 (.59; 2-4.28)** | 4.90 (.47; 4.32-6.33)** |
| 36-month SRS total (sqrt transformed) | 7.10 (2.35; 3.87-11.18)** | 4.78 (1.02; 2.65-6.93)** |
| 36-month CBCL ADHD subscale total (sqrt transformed) | 2.41 (.74; 1-3.46)** | 1.36 (.83; 0-3)** |

*p<.05, **p<.01.

CBCL = Child Behavior Checklist, EL = elevated likelihood, SRS = Social Responsiveness Scale. b = unstandardized coefficient, β = standardized coefficient.

Note: the sample included in these group comparisons are those who had complete measurement of 10-month aperiodic exponent

*Supplementary Table 2. Associations between Infant Metrics of E/I Balance and Neurodevelopmental Traits in Using Modified Social Responsiveness Scale Total*

| **Predictor** |  | **36-Month Autism Traits (SRS Brief)** | | |
| --- | --- | --- | --- | --- |
| Model 1: Main effects | b | 95% CIs | β | p |
| 10-month aperiodic exponent | 11.34 | [-8.10, 30.79] | .12 | .25 |
| EL-autism status | 5.86 | [2.34, 9.38] | .32 | <.01 |
| EL-ADHD status | 4.16 | [-.18, 8.50] | .22 | .06 |
| EL-ADHD + autism status | -3.54 | [-12.37, 5.29] | -.16 | .43 |
| Sex | -2.41 | [-5.77, .96] | -.14 | .16 |
| Age in years at 10-month visit | -.53 | [-30.55, 29.48] | -.01 | .97 |
| Number of EEG Trials at 10-month visit | -.01 | [-.05, .04] | -.03 | .75 |
| Model 2: Interaction effects |  |  |  |  |
| 24-month executive attention | 23.94 | [3.06, 44.82] | 2.73 | .03 |
| 10-month aperiodic exponent*24-month executive attention | -19.53 | [-33.09, -5.97] | -3.55 | <.01 |

The modified SRS total (SRS-Brief) excludes items that pertain to co-occurring emotional and behavioural difficulties (which are prevalent in autistic populations), thus has been proposed to be a more accurate measurement of autistic traits. See [6] for more details.

CBCL = Child Behavior Checklist, EL = elevated likelihood, SRS = Social Responsiveness Scale, b = unstandardized coefficient, β = standardized coefficient.

**References**

1. Goodman, R., et al., *The Development and Well-Being Assessment: description and initial validation of an integrated assessment of child and adolescent psychopathology.* J Child Psychol Psychiatry, 2000. **41**(5): p. 645-55.

2. Berument, S.K., et al., *Autism screening questionnaire: diagnostic validity.* The British Journal of Psychiatry, 1999. **175**(5): p. 444-451.

3. Conners, C.K. and S. Goldstein, *Conners early childhood: Manual*. 2009: Multi-Health Systems Incorporated.

4. Conners, C.K., *Conners (3rd Edition).* 2008, Toronto, Canada: Multi-Health Systems.

5. Conners, C.K., D. Erhardt, and E.P. Sparrow, *Conners' adult ADHD rating scales (CAARS): technical manual*. 1999, North Tonawanda, NY: Multi-Health Systems.

6. Moul, C., et al., *Differentiating Autism Spectrum Disorder and Overlapping Psychopathology with a Brief Version of the Social Responsiveness Scale.* Child Psychiatry & Human Development, 2015. **46**(1): p. 108-117.
